# In this issue: Volume 114, Issue 3, March 2023

**DOI:** 10.1111/cas.15408

**Published:** 2023-03-05

## 1 The cancer epigenome: Non‐cell autonomous player in tumor immunity


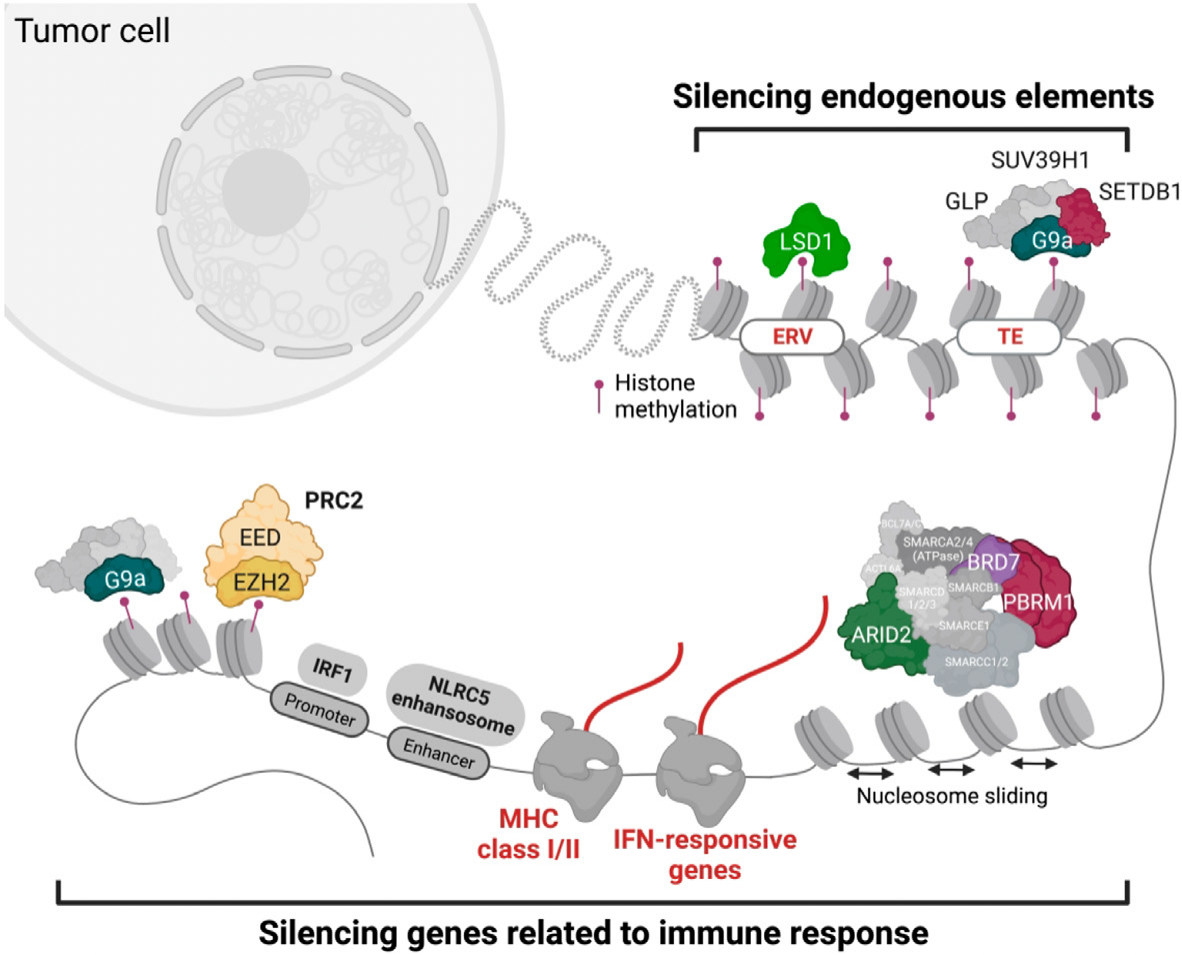


The dysregulation of epigenetic modifications, i.e., modifications that alter gene activity without changing the gene sequence, are a hallmark of cancer development. Epigenetic drivers promote cancer progression by activating or deactivating specific genes to facilitate uncontrolled cell division and preventing the detection or suppression of cancer cells by the immune system. These changes are crucial for the formation of the tumor microenvironment (TME), where the normal cell machinery is transformed to facilitate tumor formation and invasion of neighboring healthy tissue.

In this review article, Kato et al. discuss the key epigenetic changes associated with cancer progression and ways in which they can be targeted in cancer therapy. Some of the well‐known epigenetic modifications in cancer occur at the level of chromatin—fibers that constitute chromosomes. In several types of cancer, mutations are observed in histones, which are protein complexes that act as spools and wrap DNA to ensure tight packing within chromatin fibers. A notable form of mutation in this regard is observed in histone methyltransferases, a group of enzymes that modify histones.

This review focuses on two histone methyltransferases—Enhancer of zeste homolog 2 (EZH2) and Euchromatic histone‐lysine N‐methyltransferase 2 (EHMT2), also known as “G9a.” Mutant variants of EZH2 induce epigenetic reprogramming in several types of cancer, including B‐cell lymphoma, malignant melanoma, and Ewing sarcoma. By restructuring chromatin through an unknown mechanism, EZH2 mutants help cancer cells evade detection by the immune system. Similarly, G9a mutants contribute to the progression of cancers like melanomas by modifying melanocyte development pathways and assisting cells escape immune surveillance.

Recently, great strides have been made in cancer therapies that use immune checkpoint inhibitors. However, immune checkpoint blockade (ICB) therapies can be ineffective in many cases. In this article, Kato and colleagues propose a dual approach to cancer treatment with therapies targeting immune checkpoints and epigenetic modulators like EZH2 and G9a.

Finally, to aid in identifying epigenetic modulators that can be targeted, the authors propose the development of screening protocols involving DNA barcode technology and CRISPR—a valuable DNA editing and sequencing tool. By identifying optimal targets among epigenetic modulators, new drugs that prevent the formation of immune‐resistant TMEs can be developed. These drugs may minimize immune evasion by cancer cells and improve clinical response to ICB therapies.

https://onlinelibrary.wiley.com/doi/full/10.1111/CAS.15681

## 2 Probiotics enhances anti‐tumor immune response induced by gemcitabine plus cisplatin chemotherapy for urothelial cancer


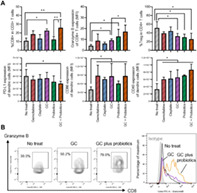


Urothelial carcinoma (UC) is an aggressive cancer with a poor prognosis. Although first‐line chemotherapy drugs like gemcitabine and cisplatin (GC) have proven effective against UC, scientists are still striving to improve drug response and achieve better patient outcomes. Previous research has shown that cancer immunotherapy may be strongly associated with gut flora or microbiota, and an imbalance in this microbial population can negatively impact a patient's overall health. A composition of these healthy gut microbes, called “probiotics” is taken as supplement to improve metabolism and immune responses. It is, however, unclear if gut microbes play an active role in modulating the anti‐tumor immune responses of GC. Moreover, the effect of composition and diversity of the gut microbiome on these responses also remains unknown.

In this study, Miyake et al. investigated the effects of GC on the gut microbiota and the impact of supplementing a probiotics mixture on anti‐tumor host immune responses. To this end, they first induced tumors in mice by injecting them with murine bladder cancer cells. The tumor‐bearing mice were then divided into two groups: one that was given GC and the other that was given both GC and probiotics. The probiotics contained a mixture of *Lactobacillus* and *Bifidobacterium* and were supplemented orally.

In the tumor‐bearing GC group, the gut microbiota showed a change in the cluster patterns of bacterial species like *Pseudoclostridium*, *Robinsoniella*, *Merdimonas*, and *Phocea*. Likewise, a comparison between GC‐treated and GC plus probiotics groups showed that the effect of gut microbiota was due to the production of short‐chain fatty acids. Changes in fecal and plasma fatty acids were also observed, which proved that oral supplementation with probiotics affects the immune and metabolic microbial changes caused by GC chemotherapy.

Mice administered GC with probiotics had lesser tumor volumes after 2 weeks of treatment. Flow cytometry analysis showed that oral probiotics could enhance anti‐tumor responses by reducing the population of cancer‐associated fibroblasts and regulatory T‐cells and activating the CD8+ T cells and dendritic cells.

In conclusion, this study proves the favorable effects of probiotics on anti‐tumor responses through the gut‐tumor immune response axis. More clinical studies will be needed to determine the full benefits of this probiotic treatment in conjunction with chemotherapy for patients with urothelial cancer.

https://onlinelibrary.wiley.com/doi/full/10.1111/CAS.15666

## 3 Cells with stem‐like properties are associated with the development of HPV18‐positive cervical cancer


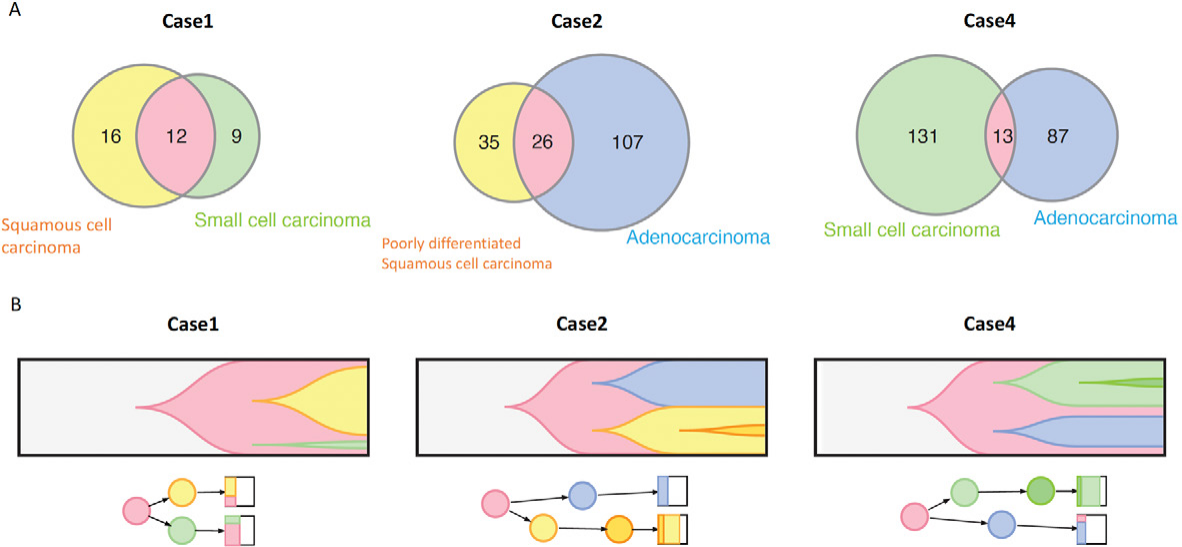


Cervical cancer is primarily caused by an infection with the human papillomavirus (HPV). While much is known about the cancer and its types, scientists are still trying to figure out how HPV‐associated cervical cancer cells or tumors differ from normal cells. This is also known as “histological differentiation,” and has long been an unsolved mystery. HPV variants or genotypes, such as HPV18, have previously been linked to the tissue level distribution of high‐risk cervical cancers. Furthermore, several hypotheses have been proposed regarding how HPV‐infected cells differentiate into cervical cancers, particularly of “mixed histological types,” that involve multiple tissue types.

In this study, Kusakabe et al. investigated the formation of cervical tumors of mixed histological types, focusing on the tumor origin, HPV variant, and tumor cell characteristics. To this end, they performed histopathological studies of tumor samples collected from cervical cancer patients. Of the 42 samples analyzed in this study, four cases had mixed tissue types. Moreover, HPV18 was present in three of the four cases. The authors used phylogenetic analysis to trace the lineage of tumor cells and found that tumors of mixed histological types were derived from the same cell.

Further genome sequencing studies revealed that the site of viral genome integration in the human genome was identical across all histological types, indicating that cell differentiation occurred only after an HPV infection. To further support this theory, RNA sequencing of these histological cancer types revealed that HPV‐derived RNA was consistent across tumor tissues.

The authors next used gene expression profiling to study the molecular mechanisms of HPV‐related cervical cancer. They found that HPV18‐associated tumors of mixed histological types were found to contain cells that could differentiate just like stem cells. These cells are also tagged “immune cold,” as they are unlikely to trigger a strong immune response.

In conclusion, the study demonstrates that once an HPV‐infected cell proliferates into a mixed cervical cancer, it differentiates into different tissue types. These findings may aid in the prevention or early detection of HPV18‐derived cervical cancers. They may also be useful in understanding the origin of mixed histological cancer types and developing effective treatments for them.

https://onlinelibrary.wiley.com/doi/full/10.1111/CAS.15664

